# Two-stage, self-cycling process for the production of bacteriophages

**DOI:** 10.1186/1475-2859-9-81

**Published:** 2010-11-01

**Authors:** Dominic Sauvageau, David G Cooper

**Affiliations:** 1 Department of Chemical Engineering, McGill University, 3610 University, Montreal, Quebec, H3A 2B2, Canada

## Abstract

**Background:**

A two-stage, self-cycling process for the production of bacteriophages was developed. The first stage, containing only the uninfected host bacterium, was operated under self-cycling fermentation (SCF) conditions. This automated method, using the derivative of the carbon dioxide evolution rate (CER) as the control parameter, led to the synchronization of the host bacterium. The second stage, containing both the host and the phage, was operated using self-cycling infection (SCI) with CER and CER-derived data as the control parameters. When each infection cycle was terminated, phages were harvested and a new infection cycle was initiated by adding host cells from the SCF (first stage). This was augmented with fresh medium and the small amount of phages left from the previous cycle initiated the next infection cycle. Both stages were operated independently, except for this short period of time when the SCF harvest was added to the SCI to initiate the next cycle.

**Results:**

It was demonstrated that this mode of operation resulted in stable infection cycles if the growth of the host cells in the SCF was synchronized. The final phage titers obtained were reproducible among cycles and were as good as those obtained in batch productions performed under the same conditions (medium, temperature, initial multiplicity of infection, etc.). Moreover, phages obtained in different cycles showed no important difference in infectivity. Finally, it was shown that cell synchronization of the host cells in the first stage (SCF) not only maintained the volumetric productivity (phages per volume) but also led to higher specific productivity (phage per cell per hour) in the second stage (SCI).

**Conclusions:**

Production of bacteriophage T4 in the semi-continuous, automated SCF/SCI system was efficient and reproducible from cycle to cycle. Synchronization of the host in the first stage prior to infection led to improvements in the specific productivity of phages in the second stage while maintaining the volumetric productivity. These results demonstrate the significant potential of this approach for both upstream and downstream process optimization.

## Background

The number of important applications for bacteriophages and their viral particles are increasing. While phage therapy was identified and initially heralded as the main application for phages [1-4], its large scale use has yet to materialize. However, in recent years, renewed interest is obvious in not only phage therapy [5-8], but also in detection and diagnostics [9-11], bacterial control [12-17] and recombinant protein production [18-20]. Moreover, bacteriophages have now been identified as important tools in many aspects of nano-medicine - such as phage display for treatment or drug discovery, gene or drug delivery or even in direct cancer treatment [21-30]. These developments have led to the re-evaluation of the potential uses of bacteriophages and to attempts to improve the methods of production.

Phages are normally produced in batch fermentations with both the advantages and inconveniences associated with this mode of operation. The phage titers obtained are elevated [31,32] and there are no issues with residence time or control strategies. However, as with all batch processes, it requires a lot of manpower, large footprints and significant capital costs. As well, the proportion of downtime to production time can be high and this limits the throughput. In general, this mode operation is neither cost-efficient nor time-efficient.

In attempting to avoid the disadvantages of the batch process, different strategies have been developed for continuous production of phages. Studies have been conducted in chemostats [32-35] and in two-stage continuous processes in which the host is grown in a first stage and fed to a second stage where the phage is being produced [32,34,36,37]. These processes allow high volumetric throughputs with smaller fermenter footprints. However, these modes of operation have also proven to have many disadvantages and limitations. One of the main issues is the difficulty in maintaining a stable, consistent, continuous system for the production of phages. Such a situation is only possible if a fine balance among the rate of phage production, the flow rate (and dilution rate) and the rate of host proliferation is maintained [32,34]. These systems are also highly dependent on the threshold population density for the maintenance of the infection [32]. Not only does this balance of rates render the process extremely sensitive to minute disturbances - creating difficulties in the control strategy and affecting the robustness of the systems - but the threshold population density is often much lower than the host densities observed in batch processes, resulting in low phages titers at the outlet [32,37,38]. Other issues often encountered in continuous processes are related to the residence time distribution [32,37]. As is the case in all continuous stirred-tanks, some host and phage particles will leave the fermenter immediately upon entrance while others may theoretically remain in the fermenter for an infinite amount of time. The first situation leads to the harvest of uninfected or non-lysed host cells, which lowers the volumetric productivity of phages. The second situation can lead to coevolution of host cells and phages. This is often observed in chemostats - in fact, chemostats are often used to study the co-evolution of host/phage systems [39-45]. The two-stage continuous systems limit to a certain extent, but do not eliminate, co-evolution [32,34].

It is possible to capitalize on the advantages of both batch (high phage titers, robustness) and continuous (high volumetric throughput, reduced downtime per production time) processes using the appropriate semi-continuous process. Examples of such processes have been reported for insect cells/baculovirus systems [46-48]. The present study focuses on the development of a cycling (semi-continuous), automated, two-stage process for the production of bacteriophages. The process developed consists of two independently-controlled stages. The first stage, in which the host cells are grown, was operated under the principles of self-cycling fermentation (SCF) [49]. SCF is a non-steady state, automated, cycling process in which a control parameter linked to cell growth (e.g. dissolved oxygen or CER) is used in a feedback control loop to trigger cycling once growth has ended. Just before the cell population enters stationary phase, half of the contents of the fermenter are removed and replaced by fresh medium. This has the double advantage of both keeping the population in exponential growth and synchronizing the population [50-54]. The second stage, in which the host is infected by the phage, was also operated in an automated cycling mode termed self-cycling infection (SCI) which, to some extent, resembles serial transfer infections [55]. The *Escherichia coli*/Bacteriophage T4 system was used as a model system to demonstrate the feasibility and implications of the system on the production of a lytic phage.

## Methods

### Bacterium, Phage and Medium

The present study was conducted with the bacterium *Escherichia coli *ATCC 11303 and the bacteriophage T4, ATCC 11303-B4. Cultures to be used as inocula were grown overnight at 37°C and 250 rpm in an incubator-shaker (New Brunswick G25, New Brunswick, NJ) to a cell density of approximately 2 × 10^9 ^cfu·mL^-1^.

The mineral salt medium used was described in a previous study [56]. Glucose (Sigma-Aldrich) was used as a carbon source at a concentration of 3 g·L^-1 ^in the cultures used as inocula, in the batch infection, in all cycles of the SCI and in the initial cycle of the SCF. The glucose concentration of the added medium used for the subsequent SCF cycles was 6-g·L^-1^.

### Optical Density, Viable Cell Density and Phage Titer Measurements

Optical density of samples from both stages of the process was measured at a wavelength of 600 nm using a spectrophotometer (Varian, Cary Bio 50).

Viable cell counts were performed for samples from the SCF stage. The samples were diluted in series and 10-µL drops were spread on plates composed of 1.5%w/v agar and 30 g·L^-1 ^tryptic soy broth. Plates were incubated overnight at 37°C and colonies were counted. The viable cell density was reported as colony forming units per ml (cfu·ml^-1^).

Measurements of free phage concentration were performed using a technique derived from the double layer method described by Maniatis *et al*. [57]. The method was described in a previous study [56]. Filtered samples (0.2µm syringe-filter) were diluted and 10µL drops were placed on soft agar containing *E. coli*. Plates were incubated overnight at 37°C. Plaques were then counted and the phage titer was reported as plaque forming units per ml (pfu·ml^-1^).

### Fermenters and Equipment

**Figure 1:**
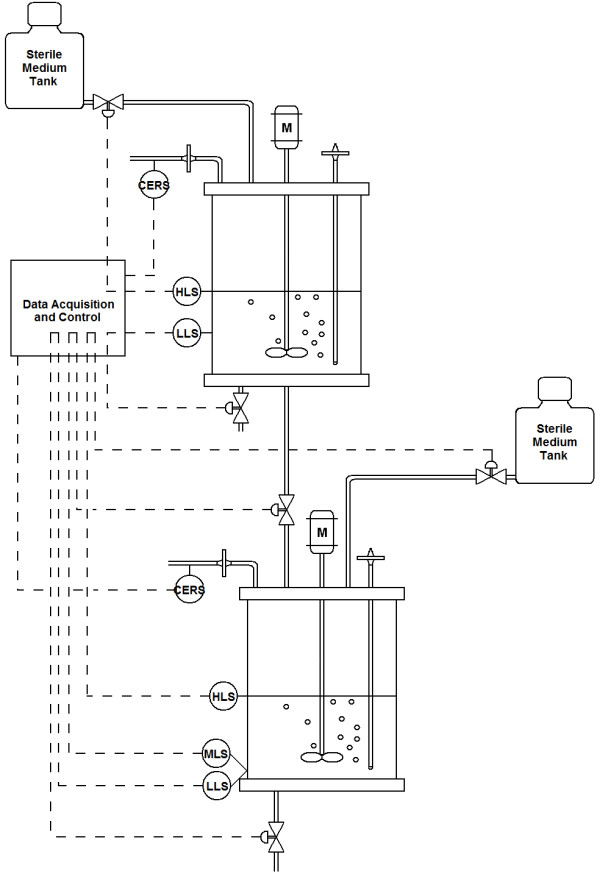
**Schematic of the self-cycling fermentation/self-cycling infection apparatus**. Level sensors are used for the control of valves (low: LLS, mid: MLS, high: HLS). The carbon dioxide sensors (CERS) are used to measure CER and as the feedback control parameter for cycling of both stages. The temperature control loops and sampling ports are not shown.

The set-up of the combined SCF/SCI process is shown in Figure 1 and includes the control connections necessary for cycling. The sensors, heaters and valves from both stages were connected to a single computer. A unique control and automation program was developed using LabView^®^. Samples were taken from sampling ports for analyses of cell density, OD_600 _or phage titer.

The SCF stage used in the present study was based on a previously described SCF set-up [54]. The only notable change made was the addition of an outlet (controlled by a valve) which allowed liquid to be passed directly to the second stage. This was equipped with an in-line isolator to avoid the infection of the host cells in the first stage by phages from the second stage. The operating conditions for the 1-L working volume were agitation: 200 rpm, temperature: 37°C and aeration: 0.5 vvm (k_L_a of 0.93 min^-1^). The carbon evolution rate (CER) was measured using an IR-spectrometry CO_2 _gas sensor (CO2-BTA, Vernier, Georgetown, ON) located after an in-line HEPA-filter at the air outlet of the fermenter. The units of CER were mol CO_2_·L^-1^·h^-1^. As was the case in the previous study, the rate of change of the CER was used as the control parameter in the feedback control loop to determine the optimal time to trigger cycling. Two electro-optic sensors (ELS-900 series, Gems Sensors) - placed at 1L and 0.5L - and current-activated valves were used to control the volume exchanges during cycling.

The fermenter used for the SCI stage was identical to the one described above with one important exception. Three electro-optic sensors were used for level control. The first was placed at 1L, the second at 0.04L and the third was placed at different volumes before the start of the process. The position of the third level sensor was set to allow the desired amount of phages to be left in the fermenter at the end of the cycles. Aeration was kept at 0.4 vvm (k_L_a of 0.84min^-1^), slightly lower than in the SCF stage. This ensured that the pressure was lower in the second vessel and prevented, along with the in-line isolator, phages from infecting the first cycle. Temperature and agitation were the same as in the SCF stage. The value of the CER and its rate of change were used as the control parameters to determine the end of the infection process and the proper time for cycling.

### Batch Infection

Batch infections were performed in the SCI stage fermenter. In such a case, the monitoring equipment (CER) and conditions (agitation, aeration) remained the same but the control system was not used because there was no cycling. The infections were started with 4 × 10^10^cfu and phages were added to the desired initial MOI.

### SCF/SCI Operation

It is important to specify that, while both stages were controlled with the same control unit (computer and software), the system could be considered as decoupled. Each stage operated independently. However, it was necessary to have communication for the short period when the SCI was cycling, when it was necessary to supply this tank with a harvest from the SCF.

The SCF stage was inoculated with 4 × 10^10^cells. The first cycle was grown as a normal batch until the conditions for cycling were met. At his point, half the contents of the vessel (0.5L) were removed to either a harvest vessel or the second stage, as required. Fresh medium was then added until the working liquid volume was recovered (1L), starting the next cycle. The cycling procedure was performed and a new cycle was started every time the conditions for cycling were met.

The SCI stage was inoculated with 4 × 10^10 ^host cells mixed with a phage stock to obtain the desired initial MOI. When the infection of the first SCI cycle was terminated (conditions for cycling were met), the following sequence of events was performed: 1) the contents of the SCI reactor were removed to a harvest vessel until the lowest level sensor of this stage was triggered; 2) the SCI stage was allowed to wait until the next SCF cycle was ready to harvest; 3) at this point, host cells were added to the SCI until a volume of 0.08L (corresponding to an inoculum of approximately 1.2 × 10^11^cells) was reached (mid-level-sensor of the SCI stage triggered); 4) any residual harvest from the SCF stage was sent to a harvest vessel until the low-level sensor of the SCF stage was triggered; 5) fresh medium was fed to refill the SCF stage to 1L (high-level sensor of the SCF stage was triggered); 6) the new SCF cycle was started; 7) fresh medium was added to the SCI stage until the high-level sensor (1L) of the SCI stage was triggered; 7) the new SCI cycle was started.

The source of phages for the infection of each SCI cycle was the volume left in the SCI from the previous SCI cycle at the end of step 1 described above. To obtain the desired initial MOI, the volume left was determined by the height of the lowest level sensor. This was adjusted prior to the beginning of operation. It was also possible to include rinsing cycles before step 2 to reduce the phage concentration and MOI if desired.

### Shake Flask Experiments

Shake flask experiments were also performed to investigate different aspects of the infections including host/phage interactions. A 0.4-mL aliquot of a suspension of the host cells, containing approximately 8 × 10^8^cells, obtained from shake flasks or from the SCF/SCI system, was mixed with 0.4-mL of a phage stock solution with a titer adjusted to obtain the desired initial MOI. The mixture was allowed to sit for 5 minutes to ensure adsorption before being added to a 500-mL shake flask containing 100 mL of medium. The shake flask was then placed in an incubator-shaker at 37°C and 250 rpm. Samples were taken periodically to measure the OD_600 _and/or the phage titer.

## Results

**Figure 2:**
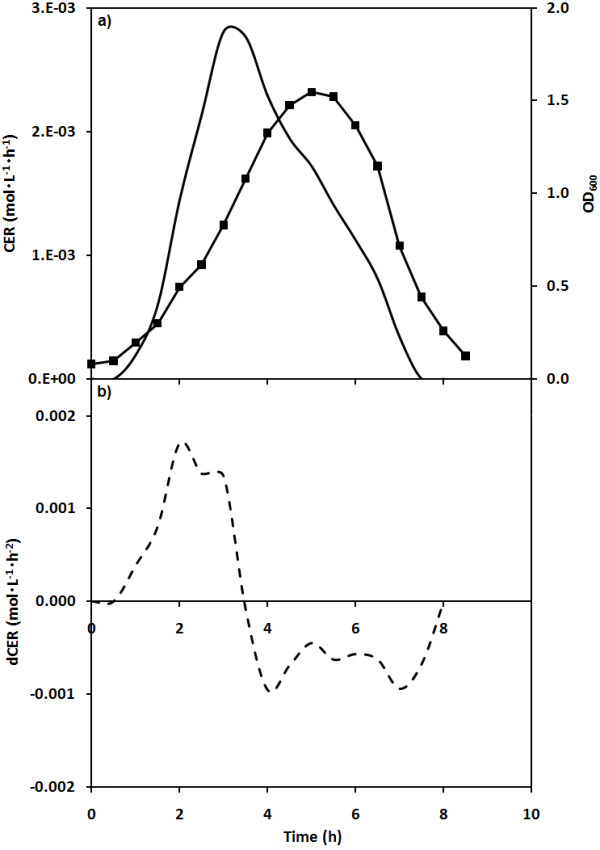
***E. coli *culture infected by bacteriophage T4 in a batch**. The CER (full line) and optical density at 600 nm (■) are shown in (a). dCER (dashed line) is shown in (b).

Figure 2a shows the patterns of OD_600 _and carbon dioxide evolution rate (CER) for a batch infection of *E. coli *by phage T4 at an initial MOI of 0.001. (The vessel was the same as that used as stage 2 during the production process.) The OD_600 _trend shows an initial increase as the host cells proliferated. A maximum was seen at 5 h after which the OD_600 _decreased until 8.2 h, indicating population-wide lysis. The CER trend shows an initial increase similar to that of OD_600_. However, the maximum was observed earlier, at 3 h, and the decrease was over by 7.8 h. Figure 2b shows the rate of change of CER - measured as the slope of the CER data (calculated over 15 minute periods) - as the infection proceeded. The values were positive up to 3.8 h after which the CER decreased and the rate of change became negative. When the infection process ended, at 8 h, the rate of change of CER returned to zero.

**Figure 3:**
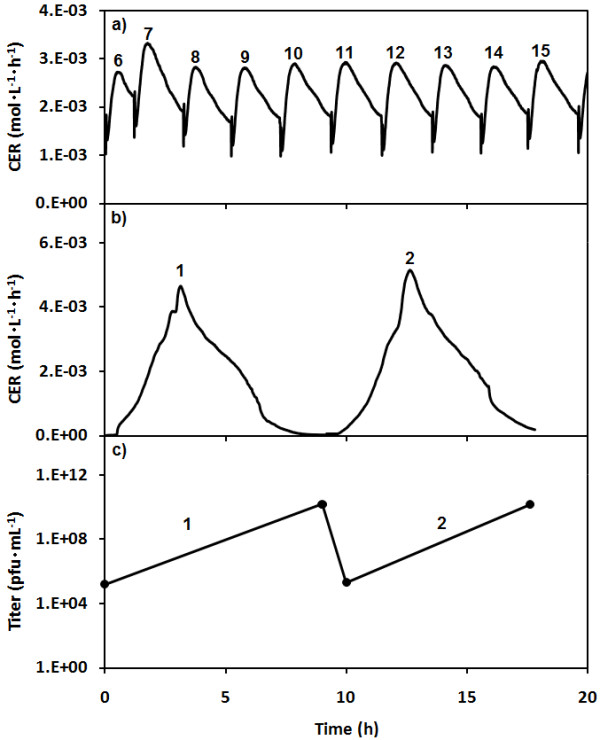
**Operation of SCF/SCI system with the SCI stage started once synchrony is established in the SCF stage**. The CER (full line) data of the SCF stage (first stage) and of the SCI stage (second stage) are shown in (a) and (b) respectively. The phage titer in the SCI stage (●) is shown in (c). The cycle numbers are shown for each cycle for their respective stage.

Figure 3 shows the CER data for an experiment that demonstrates operation of the SCF/SCI system. After synchrony had been established in the SCF (Figure 3a), cycles 5 and 10 were used to charge the SCI vessel for cycles 1 and 2, respectively (Figure 3b). The initial cell load and initial MOI of each infection cycle were 1.2 × 10^11 ^cfu and 0.001, respectively. Figure 3c shows the data for the initial and final titers of each SCI cycle.

**Figure 4:**
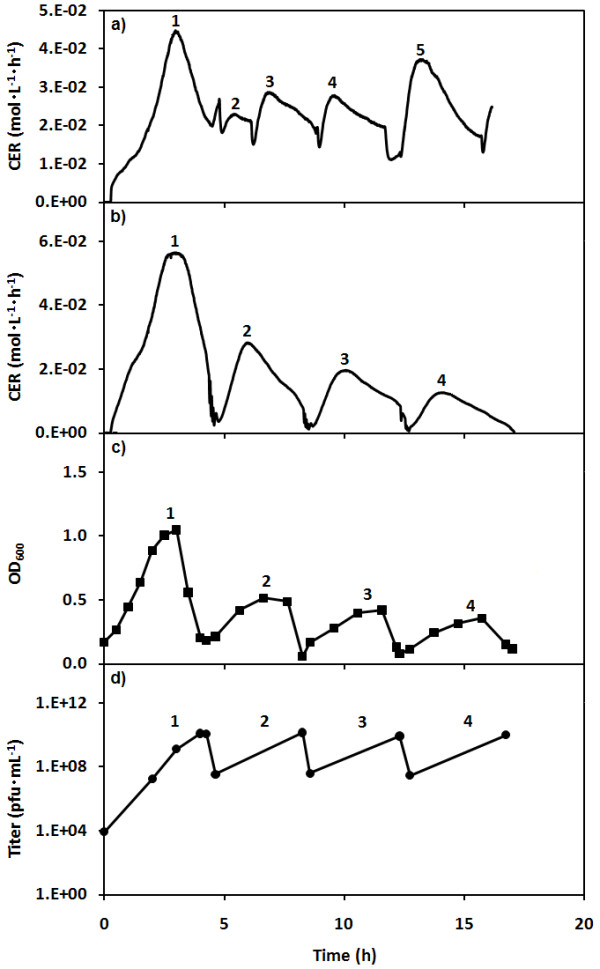
**Operation of SCF/SCI system with both stages started at the same time**. The CER (full line) data of the SCF stage (first stage) and of the SCI stage (second stage) are shown in (a) and (b), respectively. The OD_600 _(■) and the phage titer (●) in the SCI stage are shown in (c) and (d), respectively. The cycle numbers are shown for each cycle for their respective stage.

Figure 4 shows operation of the SCF and SCI systems for which both series were started at the same time using an asynchronous host population. The first infection cycle of the SCI stage was started with an MOI of 0.08. The subsequent infection cycles had an initial cell load of 1.2 × 10^11 ^cfu and an initial MOI of approximately 0.1. After the first SCI cycle, subsequent cycles were started with a charge from the SCF harvest so that SCF cycles 1, 3 and 4 were used to inoculate SCI cycles 2, 3 and 4 respectively.

In Figure 4a, the CER pattern of the SCF (first stage) starts to stabilize by the third cycle as the system tends towards synchrony. Figures 4b and 4c show the CER and OD_600 _patterns of the four SCI cycles. The duration of cycle 1 was 4.25 h, while it was 3.8 h ± 0.1 for cycles 2 to 4. While the CER and OD_600 _patterns of these cycles are all similar, they show a decrease in magnitude with each new cycle. It can be seen that the total area under the curve for each new cycle decreased for both the OD_600 _and CER data. The final phage titer of each infection cycle is shown in Figure 4d.

**Figure 5:**
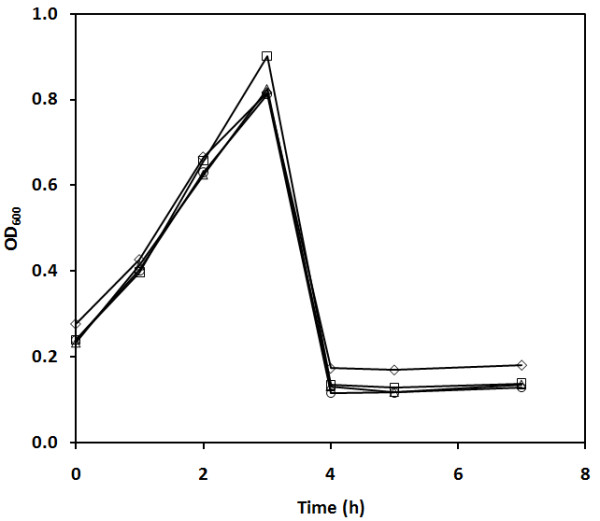
**OD_600 _during batch infections of *E. coli *infected by bacteriophage T4 harvested from different SCI cycles**. Four infections were initiated in shake flasks with 8 × 10^9^cells at an initial MOI of 0.05. The phages used were harvested from SCI cycles 1 (□), 2(◊), 3(Δ) and 4 (○).

Experiments were performed to determine if cycling had a significant effect on the virulence of phages. Figure 5 shows results from such an experiment performed with phages harvested from each of the four SCI cycles shown in Figure 4. Phages from each of these SCI cycles were used to infect 8 × 10^9^cfu of *E. coli *at an MOI of 0.05 in shake flasks. All infections were performed at the same time, using the same conditions and using samples from the same host culture as inocula. Since the sampling was done once every hour, some of the important features of the infections, such as the onset of lysis inhibition, cannot be readily identified. However, it can be seen that the four shake flask infections had exactly the same trend and the population-wide lysis was observed between 3 h and 4 h in all cases.

**Figure 6:**
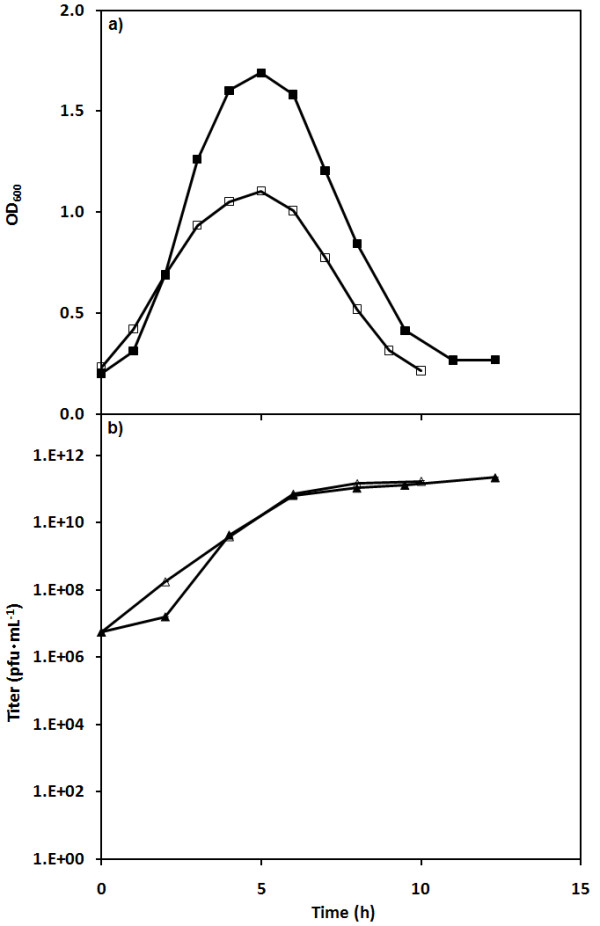
**Infections of asynchronous and synchronized *E. coli *populations by bacteriophage T4**. Infections were initiated in shake flasks with 8 × 10^9 ^cells at an initial MOI of 0.01. OD_600 _(a) and phage titer (b) are shown for asynchronous (■, ▲) and synchronized (□, Δ) host cell populations.

Shake flasks experiments were performed to compare the infection of asynchronous and synchronized host populations. The populations were taken from end-of-cycle harvests during SCF operation. The asynchronous population was taken at the end of a SCF cycle 1 in Figure 3 while the synchronized population was harvested at the end of cycle 10. (This number of cycles has been shown to be more than sufficient to establish synchrony [54].) All flasks were infected under the same growth conditions, using the same phage stock, initial cell loads (8 × 10^9 ^cfu) and initial MOI (0.01). Figure 6 shows the OD_600 _and titer data for one of these comparative experiments. It can be seen that the infection of the synchronized population led to smaller OD_600 _values and a shorter period of infection. However, the titer remained similar for both types of populations.

Two approaches were taken to assess the productivity of the system. Since OD_600 _can be used as an approximation of the cell concentration - in the case of infections, these two parameters are not proportional throughout the infection process [58] - the first approach was to approximate the specific productivity as the phage titer at the end of a SCI cycle divided by the integral of the OD_600 _data of that cycle. The units are pfu·mL^-1^·AU^-1^·h^-1 ^and this number is an estimation of pfu·cell^-1^·h^-1 ^or phages produced per cell per hour. In the second approach, the integral of the CER data of the SCI stage was used to obtain a phage productivity based on the amount of CO_2 _released (final phage titer divided by the integral of the CER data of a SCI cycle). The units are pfu·mol of CO_2_^-1^. The values of productivity and final phage titer for the SCI cycles shown in Figures 3 and 4 are collected in Table 1. No significant differences in productivity or final phage titer are seen among the cycles in Figure 3. However, while the cycles from Figure 4 show stable final titers there are increases in productivity with each cycle. Both measures of productivity are significantly greater for cycle 4 compared to cycle 1.

**Table 1:** Multiplicities of infection and phage productivity for different experiments.

Experiment	Multiplicity of Infection	Productivity	
		
		pfu·mL^-1^·AU^-1^·h^-1^	pfu·mol CO_2_^-1^
SCF/SCI (Figure 3)			
Cycle 1	0.01	-	7.59 × 10^14^
Cycle 2	0.01	-	7.00 × 10^14^
SCF/SCI (Figure 4)			
Cycle 1	0.08	4.33 × 10^9^	7.71 × 10^13^
Cycle 2	0.1	8.97 × 10^9^	2.11 × 10^14^
Cycle 3	0.1	6.98 × 10^9^	1.61 × 10^14^
Cycle 4	0.1	9.15 × 10^9^	3.17 × 10^14^
Shake flasks (Figure 6)			
Asynchronous host population	0.01	2.05 × 10^10^	-
Synchronous host population	0.01	2.68 × 10^10^	-

The results of OD_600_-derived specific productivity for infections carried in shake flasks with asynchronous and synchronized cultures are also shown in Table 1. It can be seen that the specific productivity was increased by 30% for the infection of a synchronous population relative to the asynchronous samples.

## Discussion

A problem with the application of self-cycling fermentation (SCF) has been that the original control strategy required a dissolved oxygen control that was susceptible to fouling. It has recently been demonstrated that carbon dioxide evolution rate (CER) data can also be used as the control parameter to trigger cycling and automate the SCF process [54]. A SCF modified in this way is the first stage in the process being investigated here and this showed the expected stable SCF synchronized growth pattern (Figure 3a).

This paper demonstrates that a similar control strategy based on CER can be used to control a second stage used for bacteriophage production. This SCI stage allows bacteriophages to infect host bacteria transferred from the SCF stage. Using both the CER measurements and the rate of change of the CER (dCER) data, it was possible to pinpoint the end of the population-wide lysis in the SCI, which corresponds to the end of the period of phage release. The importance of considering both parameters can be seen in Figure 2 at about 8 h, at which time the value of the CER has returned to its initial value and dCER is at 0. The d CER is a more precise control point but the first time that this parameter passed 0 was actually the point of maximum respiration and much too early to harvest the SCI; so both criteria must be met. As can be seen in Figures 3b and 4b, the control strategy was effective, robust and did not require a probe in direct contact with the cultures.

### Operation and performance of the SCF/SCI system

For a given MOI, the final phage titers obtained with the SCF/SCI system were of the same order of magnitude as those from batch productions performed with the same conditions. For example, the phage titer obtained from the SCI cycles of Figure 3c averaged 1.45 × 10^10^pfu·mL^-1^, while a batch infection initiated and performed under the same conditions yielded a phage titer of 2 × 10^10^pfu·mL^-1^. However, when using the SCF/SCI system, this production could be repeated indefinitely with no down time between harvests. This semi-continuous harvest at a high phage titer is an important advantage when compared to the low titers obtained in a continuous process [32,37,38].

The advantage over batch production was increased by the observation that the duration of each cycle in SCI operation was actually shorter than the time required for the infection in a comparable batch process. For example, the first cycle in Figure 4 is, in fact, a classical batch production and this lasted 4.25 h compared to 3.8 h for the later SCI cycles. Another example is seen in Figure 6. The infection performed with synchronized host cells was shorter than that with the asynchronous population.

In fact, the productivity of the system could be enhanced even more. The set-up used here had only a single SCI chamber and the infection cycles can be longer than the host generation cycle once stable operation is achieved, as shown in Figure 3. This is easily remedied by using harvests of host cells from a single SCF stage to provide sequentially for the required number of SCI chambers - thus optimizing the overall process.

It is important to note that the SCF and SCI stages were controlled and operated separately and these only needed to be coupled for a short period during cycling of the SCI stage. This allowed both the production of the host bacterium and its infection to be optimized separately. Reducing the interaction between the two stages also increased stability of operation relative to two-stage continuous production [32]. Typically, in the latter, the fates of both stages are closely intertwined and a disturbance in the feed flow rate, dilution rate or outlet flow rate or of conditions such as temperature, pH, growth rate or infection rate in just one stage will have a critical impact on both stages. This often leads to the termination of the process. In contrast, both stages of the SCF/SCI system operate independently and a disturbance in one cycle will not necessarily be compounded by interaction with the other stage or, if severe enough, force a shut-down of both stages. In fact, the robust nature of the SCF control allows for the system to recover from disturbances within a few cycles as seen in Figure 3 for cycles 6 and 7 and in earlier work [51,54].

### Productivity during SCF/SCI operation

Some important information regarding the average phage productivity in the SCF/SCI system can be obtained by looking at the transient behaviour observed when the system is being operated before the SCF stage is stable and cell synchrony is fully developed. The traces in Figure 4 demonstrate such an operation. The host population in the first SCF cycle had not yet achieved synchrony. In general, five SCF cycles were required for synchrony to be fully developed in the host population [54]. This means that the series of host populations used to inoculate each SCI cycle in Figure 4 had the same cell density but not the same degree of cell synchrony.

The first important observation is that even though each SCI cycle was inoculated with the same amount of host cells, the integrals of OD_600 _and of CER - which are both correlated to the overall integrated cell density - decreased with each cycle. The overall trends thus show that fewer cells were produced and less CO_2 _was released in SCI cycle n+1 relative to SCI cycle n. This means that either the trend towards synchronization of the host population or changes in the phage population caused a limitation of the host cell proliferation. Surprisingly, despite these trends, there was no significant change in the most important parameter, the final phage titer (Figure 4d). It is important to note that these transient trends were not observed for SCI cycles performed once the host cell synchrony was fully developed in the SCF stage (*e.g*. Figure 3).

Three hypotheses were investigated to explain these phenomena. The first two, which can be eliminated, are commonly seen in continuous infection processes such as chemostats. These were that the process selected for: 1) more virulent phages or 2) host cells that were more susceptible to the phage. It will be shown that instead the phenomena can be explained by the fact that 3) synchronization led to an increase in the specific productivity (phage per cell per hour), which is a consequence of burst size.

The cycling nature of the process could create a selective pressure for more virulent phages because the infection of every new cycle was initiated with phages from the previous SCI cycle. In turn, this increasing virulence would result in an increase in the rate of infection, while the rate of host cell proliferation would remain constant. This, in turn, would result in shorter infection cycles and the amplification of the more virulent phages with each cycle. Because host cells would have less time to proliferate before being infected (lower OD_600 _and CER), each new cycle would be faster and eventually a decrease in final titer would be observed. However, the increase in virulence can be dismissed. As stated above, with fewer host cells produced, the final phage titer would also decrease proportionally. This was not the case and the same final titer (volumetric productivity) was maintained throughout the cycles of the experiment (Figure 4d). Moreover, as seen in Figure 5, when batch infections were performed in shake flasks with phages harvested from each of the SCI cycles seen in Figure 4, no obvious differences were observed. The fact that the dynamics of infection were similar means that the rate of infection was also similar in all four cases. If the virulence was drastically different in one cycle compared to the others, the corresponding trends in Figure 5 would have shown shorter infection times and the OD_600 _would have been lower. These factors showed that there was no significant increase in virulence from one harvest to the next over the duration of the experiments performed.

An increase in susceptibility of the host cells could have also explained the trends in Figure 4. However, this can also be discounted. Again, an increase in susceptibility would have led to a decrease in the phage titer at the end of the cycles, which was not observed. More importantly, the SCF/SCI process could not cause selection of more susceptible hosts because the first stage was isolated from the SCI stage and the hosts were always grown in the absence of phages prior to being infected. This demonstrates an important advantage of the decoupled nature of the system - it limits the possibility of co-evolution.

As can be seen from the specific productivity results in Table 1, after the first SCI cycle in Figure 4, there is a noticeable increase in both measures of phage productivity for the subsequent cycles. Since both the OD_600 _and the CER data indicated that the total amount of cells produced decreased with each subsequent cycle but that the volumetric productivity remained constant, it follows that the specific productivity of the host cells (phages per cell per hour) must have increased with each cycle. Since the specific productivity can be used as a comparative indication of the burst size, it can be inferred that the burst size increased with each cycle as the synchrony of the host population was increasing.

To test this last hypothesis, experiments were performed in shake flasks with asynchronous and synchronized host populations infected with the same phage stock. This eliminated any possibility of differences due to other factors. These experiments showed that infecting synchronized populations led to less host proliferation and shorter infection times but equivalent final titers (volumetric productivity) (Figure 6). These are the same patterns seen in the SCF/SCI process (*e.g*. Figure 4). In fact, the specific productivity of the synchronized population was found to be greater than that of asynchronous populations (Table 1). Thus, the increase in specific productivity/burst size could be explained by the synchronization of the host cells.

Rabinovitch *et al*. [59] have shown, by infecting synchronous *E. coli *cells - obtained with a "baby-machine" - by phage T4 at different times in the cell growth cycle, that young, smaller cells have a lower burst size than older, larger cells. In their study, the authors did not report a difference in the burst size between synchronous and asynchronous cells. However, in the present study, the cells are not only synchronous (all cells at the same stage of the cell cycle) but synchronized (forced into synchrony by the cycling process) and it has been shown that the synchronization of *E. coli *cultures by SCF affects the metabolism of the cell (e.g. by lowering the doubling time) [54]. The implied increase in specific productivity seen here could then be explained by a larger cell size (due to the culture always being in exponential growth) and by the effects of synchronization. A study on the effect of synchronization on the intracellular processes and parameters involved in the infection of *E. coli *by bacteriophage T4 (e.g.burst size, lysis time, lysis inhibition) is currently underway.

A situation in which the volumetric productivity is maintained while the specific productivity is increased is obviously advantageous. Operating the SCF/SCI system would have concrete and significant impacts on both the up-stream and the down-stream processes because it would be possible to obtain a higher phage yield per operating volume, which makes the production cheaper and more labour-efficient. The results presented here also show that synchronized host cells will yield more phages per cell so that fewer cells are required to obtain a given titer. Therefore, the proportion of cell debris (membrane, host proteins, etc.) to phages would be reduced, facilitating the recovery of the phages and lowering the costs associated with downstream processing.

## Conclusions

Production of bacteriophage T4 using the SCF/SCI process has been shown to have many advantages over both batch and continuous production methods. The system provided semi-continuous harvests, which reduced the proportion of down-time relative to production time. Each harvest had titers at least as high as those from batch infections carried out under similar conditions. The independent operation of both the first and second stages reduced the chances of co-evolution and there was no evidence for the selection of either more susceptible hosts or for more virulent phages.

The synchronization of the host cell populations in the SCF stage improved the specific phage productivity of the host cells in the SCI stage. This resulted in a reduction of the time of infection and the yield, as indicated by integrating OD_600 _or CER, when compared to batch infections. The use of the SCF/SCI system can reduce the process time and the costs associated with both the upstream and downstream processes.

## Competing interests

The authors declare that they have no competing interests.

## Authors' contributions

DS conceived the study, designed and performed the experiments and wrote the manuscript. DGC supervised the study, participated in the design of experiments and edited the manuscript. Both authors read and approved the manuscript.
